# Mechanical Properties and Energy Absorption Characteristics of Additively Manufactured Lightweight Novel Re-Entrant Plate-Based Lattice Structures

**DOI:** 10.3390/polym13223882

**Published:** 2021-11-10

**Authors:** Sultan Al Hassanieh, Ahmed Alhantoobi, Kamran A. Khan, Muhammad A. Khan

**Affiliations:** 1 Mechanical Engineering Department, Khalifa University of Science and Technology, Abu Dhabi 127788, United Arab Emirates; 100049708@ku.ac.ae; 2 Aerospace Engineering Department, Khalifa University of Science and Technology, Abu Dhabi 127788, United Arab Emirates; 100045746@ku.ac.ae; 3 Advanced Digital & Additive Manufacturing Center, Khalifa University of Science and Technology, Abu Dhabi 127788, United Arab Emirates; 4 School of Aerospace, Transport and Manufacturing, Cranfield University, College Road, Cranfield MK43 0AL, UK; Muhammad.A.Khan@cranfield.ac.uk

**Keywords:** additive manufacturing, plate lattice, stereolithography (SLA), compression response, resin, energy absorption

## Abstract

In this work, three novel re-entrant plate lattice structures (LSs) have been designed by transforming conventional truss-based lattices into hybrid-plate based lattices, namely, flat-plate modified auxetic (FPMA), vintile (FPV), and tesseract (FPT). Additive manufacturing based on stereolithography (SLA) technology was utilized to fabricate the tensile, compressive, and LS specimens with different relative densities (ρ). The base material’s mechanical properties obtained through mechanical testing were used in a finite element-based numerical homogenization analysis to study the elastic anisotropy of the LSs. Both the FPV and FPMA showed anisotropic behavior; however, the FPT showed cubic symmetry. The universal anisotropic index was found highest for FPV and lowest for FPMA, and it followed the power-law dependence of ρ. The quasi-static compressive response of the LSs was investigated. The Gibson–Ashby power law (≈ρ^n^) analysis revealed that the FPMA’s Young’s modulus was the highest with a mixed bending–stretching behavior (≈ρ^1.30^), the FPV showed a bending-dominated behavior (≈ρ^3.59^), and the FPT showed a stretching-dominated behavior (≈ρ^1.15^). Excellent mechanical properties along with superior energy absorption capabilities were observed, with the FPT showing a specific energy absorption of 4.5 J/g, surpassing most reported lattices while having a far lower density.

## 1. Introduction

Lightweight engineering cellular materials are being extensively used and investigated in a wide range of industries such as the aerospace, biomedical, and transportation industries. In addition to the benefits brought about by light weighting, the dire need for materials with mechanical properties customizable by design was the motive behind the development of the so-called material concerned sub-field “Architected Cellular Materials (ACMs)” or referred to as lattice structures (LSs) [1]. LSs are formed by arranging unit cells made of struts, shells, and/or plates into a three-dimensional periodic array. Compared to solid continuum materials, LSs, although lower in density, have high specific strengths and superior energy absorption characteristics, making them widely used in modern-day applications [2,3,4]. LSs properties are mainly driven by unit cell topology, scale, and the constituent material’s properties. Their customizability also allows for the development of application-specific materials [5,6,7].

With the advent of additive manufacturing technologies, architectures previously unimaginable became possible, allowing for the design and fabrication of complex LSs with multifunctional structural capabilities, displaying unprecedented mechanical performances [8,9]. Various additive manufacturing processes currently exist that allow for the fabrication of various materials such as metals, ceramics, polymers, and composites [10,11]. A particular area of interest concerns those LSs whose members deform in a stretching-dominated rather than a bending-dominated response mode. The stretching-dominated lattices exhibit stiffness and strength properties that scale linearly with density, ρ, while strength and stiffness properties scale as ρ^3/2^ and ρ^2^ respectively for bending-dominated structures [1]. To fabricate these complex centimeter-scale-sized lattice structures, several additive manufacturing techniques have been developed, such as fused filament fabrication (FFF) [12], selective laser sintering (SLS), direct laser writing (DLW) [13,14,15], selective laser melting [16,17], and direct ink writing [18].

Cellular materials can be divided into two categories, periodic lattice structures and random stochastic foams [19]. In most cases, the periodic lattice structures contain periodically repeating unit cells with mechanical properties that can be easily customized. Random stochastic foams, the first generation of manmade porous–isotropic materials, have been widely used in impact energy absorption applications and elastic cushioning applications [1,20,21]. Comparing the two cellular structures shows that the mechanical properties of periodic lattice structures are superior to their stochastic counterparts. This is due to the fact that unit cell elements in periodic lattice structures stretch/compress under both static or dynamic loading. In contrast, stochastic foams exhibit a bending-dominated deformation mode [22].

Investigation into the usage of cellular materials for energy absorption has become rampant, with researchers realizing the potential behind utilizing LSs to improve the energy absorption of structures. Generally, honeycombs made using AM are used for energy absorption purposes; as a result, they have been widely investigated [23,24,25,26,27]. Triply periodic minimal surface (TPMS)-based lattices are also currently under the spotlight and have been recently investigated [28]. The more rudimentary truss-based lattices suffer from low manufacturability and suffer from the presence of stress concentrations upon loading and unloading [29,30,31], unlike TPMS-based lattices. The TPMS-based lattices not only lack stress concentrations but are also easier to fabricate at different scales due to their parametric nature [32]. Various studies have been conducted to investigate the energy absorption capabilities of TPMS-based lattices. and many have reported promising results [33,34,35]. Another type of LS currently gaining attraction is plate-based lattice structures [36,37], as their superior stiffnesses makes them excellent candidates for load-bearing applications. Berger et al. [38] showed that plate-based lattice structures were capable of approaching the upper Hashin–Shtrikman bounds: the theoretical limits of a composite material’s modulus and strength. In fact, Crook et al. [39] recently fabricated cubic-octet plate lattices that have reached the upper Hashin–Shtrikman bounds. Plate-based lattices also seemingly excel when it comes to specific energy absorption. Numerical simulations done by Tancogne-Dejean et al. [40] comparing truss and plate-based lattice structures of the same mass and material showed that the specific energy absorption of plate-based lattice structures is around 45% greater than that of truss-based lattice structures. They further showed the superiority of the energy absorption of plate lattices by reporting that a 316L stainless steel plate LS achieved a 30% higher specific energy absorption compared to a titanium structure. Various papers have used numerical/theoretical methods to investigate the effective properties of lattice structures and have validated them experimentally [2]. Kladovasilakis et al. [41] investigated the effective mechanical behaviors of four lattices by experimentally testing them under compression and then subsequently developing hyper-elastic FE models to predict the lattice’s behaviors.

Due to the nature of the field of cellular materials, novel architected materials with a myriad of interesting properties are constantly emerging. In this paper, three novel plate-based hybrid LSs are design, fabricated, and then had their mechanical performances characterized. Each of the three novel architectures are fabricated with 5%, 10%, 15%, and 20% relative densities. All the lattices are tested under quasi-static compression loading and then, the obtained data are analyzed and discussed in terms of their mechanical properties, such as Young’s modulus, strength, toughness, and specific energy absorption performance. The elastic anisotropic index has been analyzed, and some interesting conclusions have been presented.

## 2. Design of Lattice Architecture

**Figure 1:**
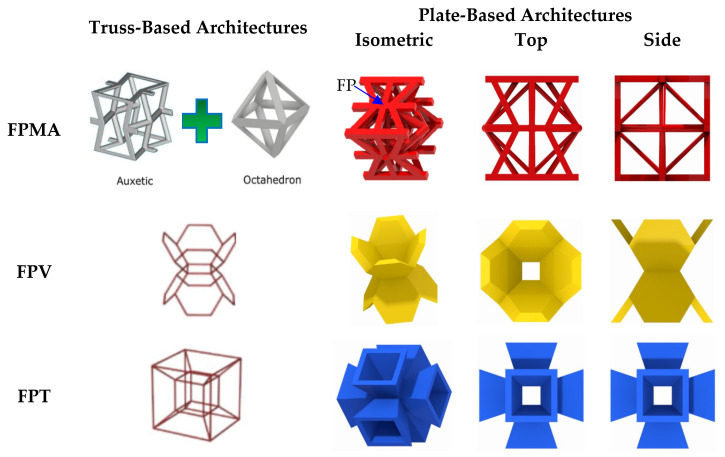
Novel re-entrant plate-based lattice structures.

The first novel design, the flat plate modified auxetic (FPMA) unit cell, is inspired by the traditional auxetic and the octahedron cell design [42]. To incorporate the octahedron unit cell into the center of the auxetic unit cell, a thin rectangular flat plate structure has been added to four of the unit cell faces to support the corners of the octahedron, as illustrated in Figure 1. To determine the dimensions of the novel unit cell, a constant thickness principle was adopted, where the flat-plate structures are of constant thickness throughout, and the trusses of the octahedron were of a constant diameter equal to 1.2 times the flat plate thickness to aid the octahedron structure.

Next, the second and third novel flat-plate tesseract (FPT) and vintile (FPV) unit cells were developed from the two traditional truss-based unit cells illustrated in Figure 1. The development of such unit cells will help directly compare and explore the mechanical properties of flat-plate structures instead of truss-based structures. Joining the truss regions with flat plates and maintaining a constant relative density meant that the plates were extremely thin, limiting their effectiveness. To account for this and help achieve a greater plate thickness for the same relative density, the angle at which the flat plates were placed was reduced to limit the extension of each plate, allowing for a thicker plate. All resultant plate-based lattices have been observed to have a re-entrant feature, which is potentially good for energy absorption behavior [1].

## 3. Methodology

### 3.1. Fabrication and Characterization

Additive manufacturing based on a VAT photopolymerization process, as defined by ISO/ASTM 52900 [43], was adopted to fabricate all specimens. The VAT photopolymerization process used is the SLA process [44]. In SLA technology, liquid resin is exposed to an ultraviolet (UV) laser that polymerizes the resin layer by layer, fabricating the 3D physical models. In this study, we employed the FormLab 3 printer and used the “Durable” resin provided by Formlabs, Inc., Somerville, MA, USA. Durable is a liquid photopolymer, which is usually comprised of a liquid mixture that is made of acrylate oligomers/monomer, methacrylated oligomers/monomer, and Photoinitiators [45]. The material has a density of ≈1.20 g/cm^3,^ low modulus, and high impact strength and failure strain, allowing for a more ductile response.

**Figure 2:**
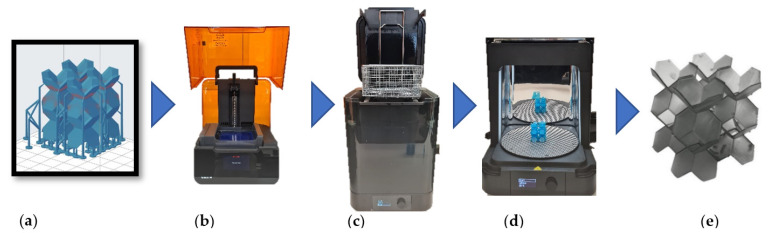
Fabrication process. (**a**) slicing software, (**b**) FormLab 3© printer, (**c**) FormLab Wash, (**d**) FormLab Cure, (**e**) final product.

In this study, we considered four different relative densities: 5%, 10%, 15%, and 20%. We first created 3D computer-aided design (CAD) models of the architected LSs using the Creo Parametric 7.0 software. The CAD files were exported as a stereolithography (STL) file format and then sliced using the PreForm 3D printing software and transferred to the FormLab 3 printer. In this printer, a high-powered ultraviolet (UV) laser polymerizes the UV-curable resin layer by layer. By varying the optical size of the UV laser, the layer resolution can be controlled in the range of 25 to 300 μm. Various lattices had a considerable number of overhangs as shown in Figure 2a; these overhangs were addressed by simply adding more support material where needed. All the fabricated lattices were printed with a 150 μm XY plane feature resolution. Two samples of each relative density were fabricated. After printing, all samples were cleaned in Formlab’s Form Wash machine and left to cure in Formlab’s Form Cure machine for two hours at a temperature of 60 °C. Then, the support material supporting the overhangs was removed using flush cutters. The step-by-step-fabrication process is illustrated in Figure 2.

**Figure 3:**
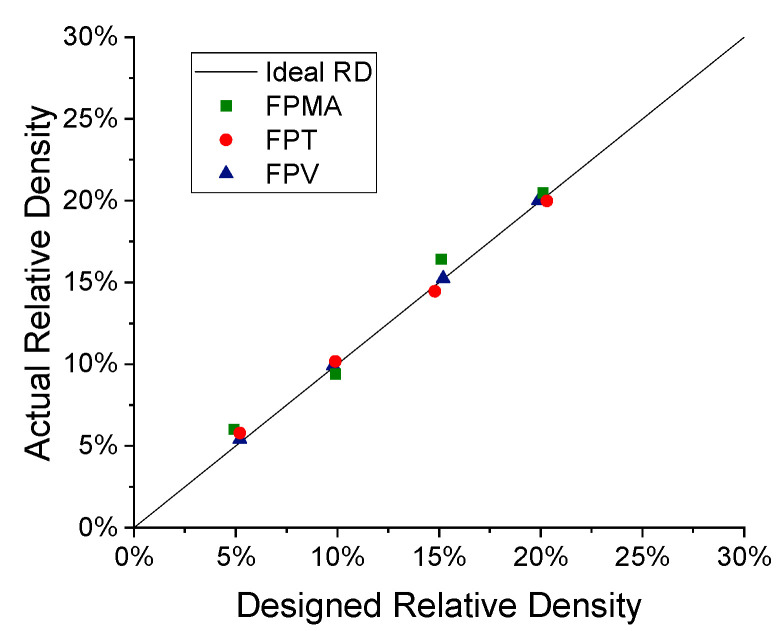
Actual vs. designed relative density.

Then, all the fabricated lattices were weighed using a METTLER TOLEDO ME204 by Mettler Toledo, Zurich, Switzerland, which is a high-precision scale with a resolution of 0.1 mg. The masses were recorded in air and utilized in calculating the relative densities. The overall dimensions of the lattices were also recorded and then used in stress and strain calculations. Afterwards, the CAD models were used to obtain the design masses, which were then utilized to compute the designed relative density. Then, Figure 3 was constructed, showing the deviation of the actual relative density and the designed relative density. All the lattices have relative densities close to their ideal relative densities, and the slight deviations may result from the presence of voids, under-cured regions, dimensional inaccuracies, undeveloped features, etc. The FPMA shows the greatest deviation from ideality due to the large amount of support material, some of which was very difficult to remove. This causes a slight increase in the measured mass of the lattice and consequently an increase in the calculated relative density.

It is emphasized here that the complex geometrical and overhanging features of the LSs limit the printability of the walls to a minimum thickness of 0.2 mm. A unit cell size of 20 mm × 20 mm × 20 mm was considered to ensure that all the samples can be printed given the minimum thickness limit of 0.2 mm. The unit cells’ minimum wall thicknesses are highlighted in Table 1. In order to obtain the properties of the parent material to be used in further analyses, three tensile samples and three compressive samples were fabricated. The tensile and compressive samples were fabricated according to ATSM D638 and ATSM D695, respectively. The specimens were fabricated using the same print parameters as was used to fabricate the lattices to ensure the applicability of the obtained results.

### 3.2. Parent Material Mechanical Testing

**Figure 4:**
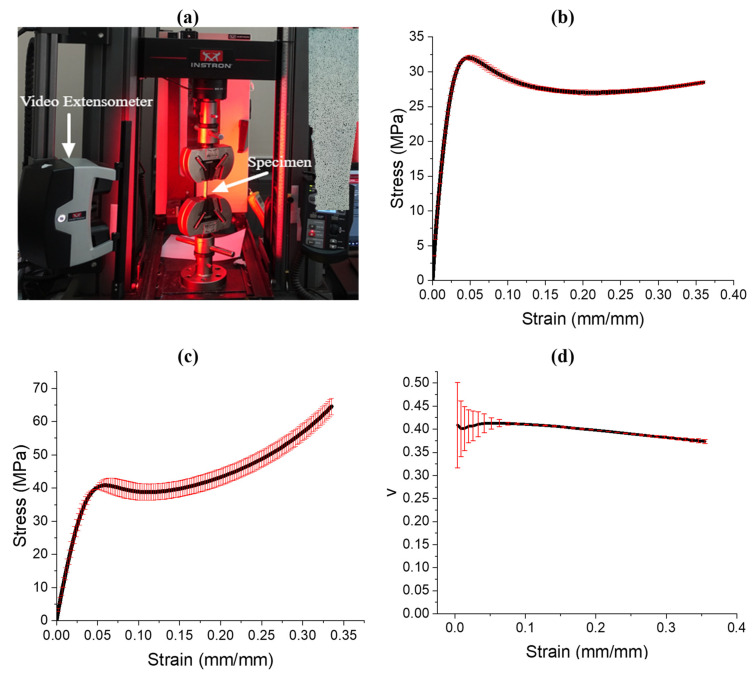
Mechanical testing, (**a**) tensile testing setup, (**b**) tensile stress–strain curve, (**c**) compression stress–strain curve, (**d**) Poisson’s ratio vs. axial strain. The error bars are colored in red.

All the compression and tensile testing was performed on the Instron 5969 Universal Testing System with a load cell capacity of 50 kN. The tensile tests’ experimental setup is show in Figure 4a. A speckle pattern was applied on tensile coupons, as shown in the inset of Figure 4a. Digital Image Correlation (DIC) was utilized to obtain the full-field displacement and strain. The tensile testing of the specimens was performed perpendicular to the printing direction with a crosshead velocity of 2.5 mm/min at room temperature until the failure of the sample. The average stress–strain curves along with the error bars for both the tensile and compressive specimen are shown in Figure 4b,c, respectively. The results of Poisson’s ratio obtained from the DIC results are also shown in Figure 4d.

From the obtained plots, the tensile and compressive moduli, the ultimate strength, the yield strength, and the constant Poisson’s ratio were obtained. The error bars are colored in red. The compressive and tensile moduli, defined as the slopes of the linear region on the stress–strain curves, were obtained as 1.22 GPa and 1.10 GPa, respectively. The compressive and tensile strengths, defined as the first inflection in the stress–strain curve, were obtained as 40.86 MPa and 32.0 MPa, respectively. The tensile yield strength, obtained using the 0.2% offset method, was obtained as 13.27 MPa. Poisson’s ratio, defined as the lateral strain divided by the longitudinal strain, was obtained as approximately 0.397.

Both the tensile modulus and strength reported in this study are comparable to those provided by the manufacturer. The manufacturer has reported a tensile modulus of 1.26 GPa and an ultimate tensile strength of 32.0 MPa [46]. The minor difference in modulus may be attributed to a variation in print parameters or post-curing conditions.

### 3.3. Homogenization of Lattice Structures

In many engineering applications, it is important to understand the anisotropy of a structure and to recognize its weakest and strongest directions. This is particularly crucial for applications involving load bearing and energy absorption, as an anisotropic lattice will not function as effectively in all its orientations. Anisotropy though, is not always a negative attribute to have, because if the expected loading direction is known, then aligning the lattice’s stiffest direction with the loading direction would be a suitable option.

The linearized macroscopic behavior of LSs can be described using the generalized Hooke’s law relating the effective stresses (${\bar{\sigma }}_{ij}$) and strains (${\bar{\varepsilon }}_{kl}$), i.e., ${\bar{\sigma }}_{ij}={\bar{C}}_{ijkl}{\bar{\varepsilon }}_{kl}$. Here, ${\bar{C}}_{ijkl}$ are the components of the fourth-order elasticity tensor. Depending on the symmetry of the LSs, the homogenized components of the ${\bar{C}}_{ijkl}$ can be defined as a function of scalar valued independent elastic moduli. For example, isotropic, cubic, orthotropic, and generalized anisotropic materials require 2, 3, 9, and 21 elastic constants.

LSs have a defined repeating pattern; therefore, the unit cell homogenization method can be adopted by choosing a representative volume element (RVE) or unit cell and applying periodic boundary conditions (PBCs) [47,48]. The complete characterization of the linear elasticity tensor can be realized using this homogenization approach. The results of the effective properties obtained from the homogenization process represent the macroscopic response of LSs.

**Figure 5:**
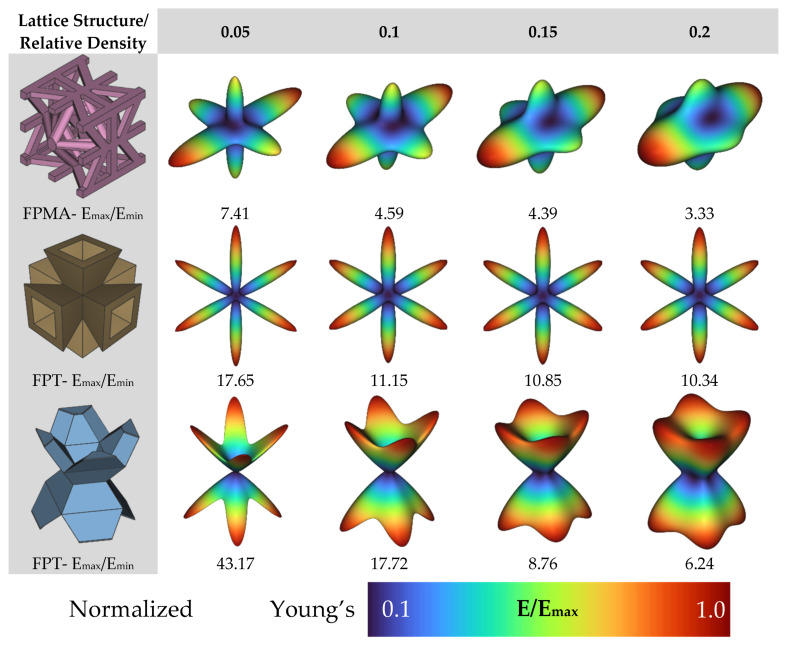
Homogenized elastic moduli in all directions.

In this study, a finite element-based numerical homogenization procedure was used to calculate the effective stiffness matrix of the LSs based upon the properties of the base material obtained in Section 3.2 and topological configuration in the RVE [49,50,51,52]. Moreover, the elastic anisotropic analysis of the LSs with different densities were performed through plotting the elastic moduli surface as a function of direction in three-dimensional space. The nTopology software [53] was used to homogenize the proposed three novel lattices and to obtain their respective stiffness matrices. In nTopology, a CAD model of each of the lattices’ unit cells were imported. The material was assumed to follow linear elastic behavior and the elastic modulus and Poisson’s ratio obtained from the tests discussed in Section 3.2. A triangular surface mesh with an edge length of 10 mm was first created from the imported CAD body; then, a tetrahedral volume mesh was generated. The nTopology “Homogenize Unit Cell” Block was used to conduct the homogenization. Figure 5 shows the proposed architectures and their elastic moduli surface as a function of direction in three-dimensional space depicting the ratio of the local Young’s modulus to the maximum Young’s modulus in every direction. Tancogne-Dejean et al. [40] proposed E_max_/E_min_ as a measure of anisotropy of the LS and found the value of 18.2 for the BCC LS at a relative density of 0.1. Here, we observed that the relative modulus of the FPV in the [1 0 0] direction was extremely small, giving E_max_/E_min_ = 43.17 at a relative density of 0.05. In contrast, the relative modulus of the FPMA in the [1 0 0] direction was reasonable, giving E_max_/E_min_ = 3.33.

The results of the homogenization process showed that the FPT belongs to the cubic symmetric system, meaning that the stiffness matrix takes on the form shown below, where $\bar{C}$_11_ = $\bar{C}$_22_ = $\bar{C}$_33_, $\bar{C}$_12_ = $\bar{C}$_13_ = $\bar{C}$_23_, and $\bar{C}$_44_ = $\bar{C}$_55_ = $\bar{C}$_66_, with the remaining constants equaling zero.

(1) $$C=\left[\begin{array}{cccccc} {\bar{C}}_{11} & {\bar{C}}_{12} & {\bar{C}}_{12} & 0 & 0 & 0\\ {\bar{C}}_{12} & {\bar{C}}_{11} & {\bar{C}}_{11} & 0 & 0 & 0\\ {\bar{C}}_{12} & {\bar{C}}_{12} & {\bar{C}}_{11} & 0 & 0 & 0\\ 0 & 0 & 0 & {\bar{C}}_{44} & 0 & 0\\ 0 & 0 & 0 & 0 & {\bar{C}}_{44} & 0\\ 0 & 0 & 0 & 0 & 0 & {\bar{C}}_{44} \end{array}\right]$$

Since the FPT belongs to the cubic symmetric system, the Zener ratio ($Z=2{\bar{C}}_{44}/\left({\bar{C}}_{11}-{\bar{C}}_{12}\right)$) may be used to measure the degree of elastic anisotropy in a cellular material. The Zener ratio of 1 indicates an isotropic lattice and larger deviation from unity demonstrating more elastic anisotropy. The Zener ratio of the FPT is calculated as 0.039, 0.063, 0.067, and 0.072 for relative densities of 5, 10, 15, and 20%, respectively.

The FPMA and FPV show anistropic behavior, and since the Zener ratio may only be used for lattices with cubic symmetry, the Universal Elastic Anisotropy index ($A^{U}$) will be used with the FPMA and the FPV. Ranganathan and Starzewski [54] proposed the following relation for the universal elastic anisotropic index.

(2) $$A^{U}=5\frac{G^{V}}{G^{R}}+\frac{K^{V}}{K^{R}}-6\geq 0$$

where $G^{V}$ and $G^{R}$ are the Voigt and Reuss shear modulus estimates, and $K^{V}$ and $K^{R}$ are the Voigt and Reuss bulk modulus estimates. An $A^{U}=0$ indicates isotropic LSs, while departure from zero defines the degree of elastic anisotropy that exists in the LSs while accounting for both the shear and bulk contributions.

**Figure 6:**
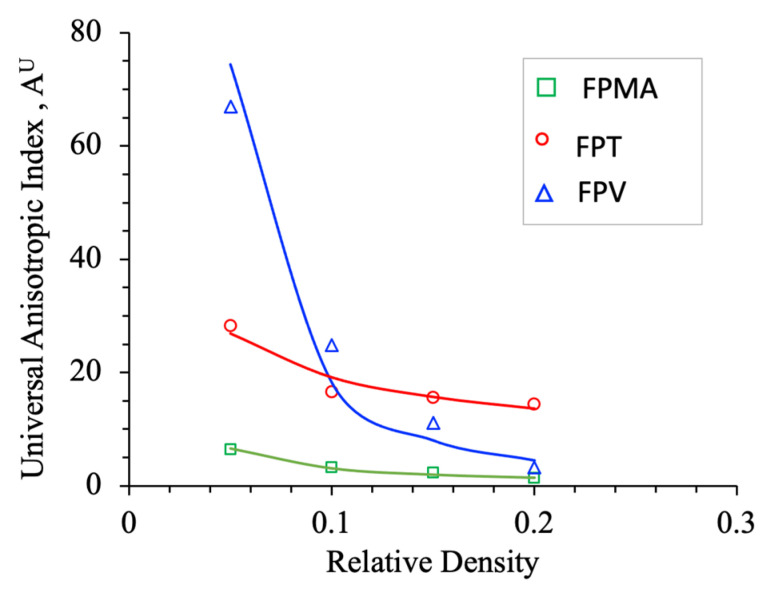
Universal Anisotropy Index of the three proposed plate-based lattice structures.

The relative density of the LSs provides a subtle control parameter to describe the universal elastic anisotropy. In this study, the function $A^{U}=B\rho^{m}$ is used to describe the universal anisotropy index relation as a function of relative density. Figure 6 shows the $A^{U}$ for the three proposed plate-based lattice structures, exhibiting a decreasing trend with increasing relative density. The FPV lattice has $A^{U}=74.4$ at ρ=0.05, which decreased rapidly to $A^{U}=4.46$ at ρ=0.2, which shows that the anistropy of FPV is very sensitive to relative density. In contrast, the $A^{U}$ of both FPMA and FPT showed a mild dependence on the relative density. The power law fitting for FPV, FPT, and FPMA is $A^{U}=0.17\rho^{-2.03}$, $A^{U}=6.2\rho^{-0..49}$, and $A^{U}=0.25\rho^{-1.09}$, respectively. The power law relations can be used to specify a relative density to provide a required level of elastic anisotropy from the proposed LSs.

### 3.4. Lattice Structure Mechanical Testing

**Figure 7:**
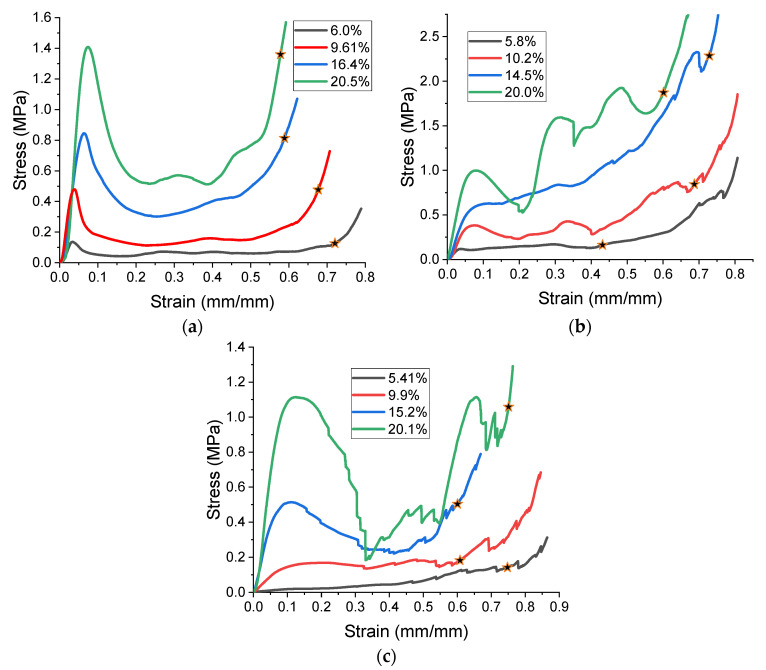
Stress–strain curves, (**a**) flat-plate modified auxetic, (**b**) flat-plate tesseract, (**c**) flat-plate vintile.

Then, all the fabricated lattices were loaded under compression in a direction perpendicular to the print direction using the Instron 5969 Universal Testing System at a constant strain rate of 0.001/s and the test was stopped when evident densification was observed. The samples were placed in the center of the machine’s plates to ensure uniform loading and to obviate moments that may arise due to sample misalignment. Figure 7 shows the average stress–strain curves of all the lattices at different relative densities, and the star symbol on each of the stress–strain curves indicates the onset of densification as per the efficiency curve criteria [55]. Pictures of the lattices at various strain levels were taken and shown in Table 2.

## 4. Results and Discussion

Under low strain rate compression, cellular structures exhibit three stages of deformation [1]. The first stage is the elastic region, from which the elastic properties are calculated. Then, the peak stress indicates the transition to the second stage, the plateau stage. The failure response of a cellular structure after the peak stress is highly dependent on its architecture, and different architectures generally exhibit different responses at the plateau stage. The final stage is densification, which is characterized by a rapid increase in the stress.

A general trend to observe in all the three lattices is the proportional relation between mechanical properties and relative density. It is also worth noting that the FPT shows the most stable response, with an average increasing stress up until the onset of densification. Beginning with the FPMA, an expected initial linear response is seen until the peak stress is reached, after which a sudden drop in the stress is observed. The FPMAs show the same trend past their peak stress, which may be attributed to the manner at which the lattices fail. At all four relative densities, failure occurs when either the trusses of the octahedron or the trusses of the auxetic honeycomb buckle, which is accentuated in the red circle, as seen in the first row of Table 2.

However, the stress–strain curves of the FPT and FPV show unorthodox behavior that is not commonly seen in other common architectures. The FPV shows the largest increase in both modulus and peak stress with an increase in relative density, albeit at the expense of a stable response after the peak stress. This loss of stability at higher relative densities may be readily explained by observing the failure mechanisms of the FPV at the four different relative densities. At the lower relative densities, the lattice experiences a more bending dominated failure mode, as shown in the red circle, which is primarily due to local buckling, as shown in the second row of Table 2. However, as the relative density increases, the lattices increase in stiffness and rather than failing by local buckling, they fail by brittle fracture, and an example in the red circle is shown in the third row of Table 2. This behavior, as a result, causes a more catastrophic failure mode as the relative density increases.

Lastly, all the FPTs exhibit relatively similar failure behavior at the lowest three tested relative densities but behave in a noticeably different manner at 20% relative density. This is explicable by looking at their failure mechanisms. At 5% relative density, the FPT crushes uniformly, as shown in the fourth row of Table 2, providing a stable failure response. The response is slightly altered at relative densities of 10% and 15% as the middle vertical members, which are circled red, remain rigid, but push into the lattice, and with increasing strain push further down, increasing the resistance to deformation and increasing the lattice’s stiffness. This phenomenon may be seen in the fifth row of Table 2. However, at 20% relative density, the middle vertical members are the first to crush, and once completely crushed, the two horizontal plates come in contact, stiffening the lattice as seen in row six of Table 2. This trend repeats until densification. These unique responses prompted the team to investigate the behavior of these novel lattices when fabricated from different materials, using different technologies, which is the group’s current work.

**Figure 8:**
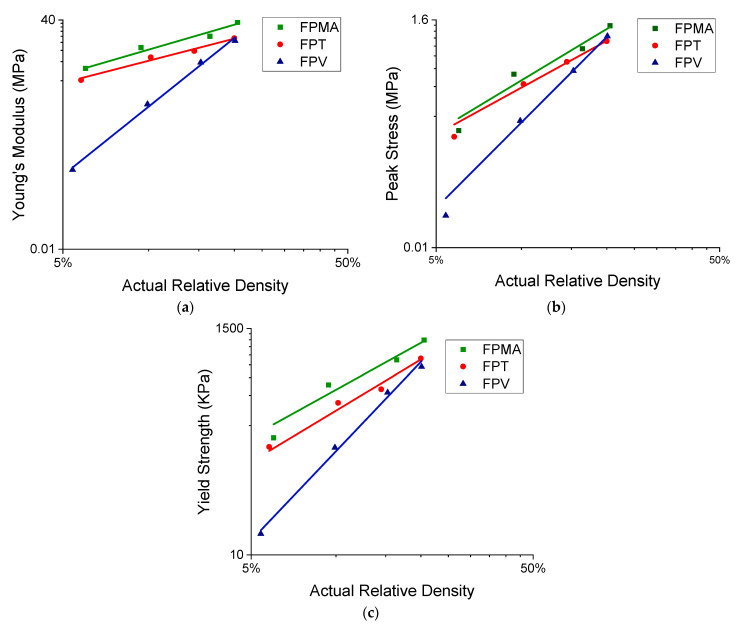
Deduced mechanical properties. (**a**) Young’s modulus vs. relative density, (**b**) peak stress vs. relative density, (**c**) yield strength vs. relative density [56,57].

The compressive modulus, peak stress, toughness, and specific energy absorption were plotted on a log–log scale and fitted with a power law of the form ∅_cellular_ = Cρ^n^, where ∅_cellular_ is the mechanical property and ρ is the relative density of the lattice structure. Figure 8a,b show the variation of Young’s modulus and peak stress with change in relative density. Young’s modulus was obtained by finding the slope of the linear region in the stress–strain curves. The peak stress is defined as the first inflection point in the stress curve. Figure 8c shows the yield strengths of all the lattices at different relative densities, where the yield strength is defined using the 0.2% offset method.

**Figure 9:**
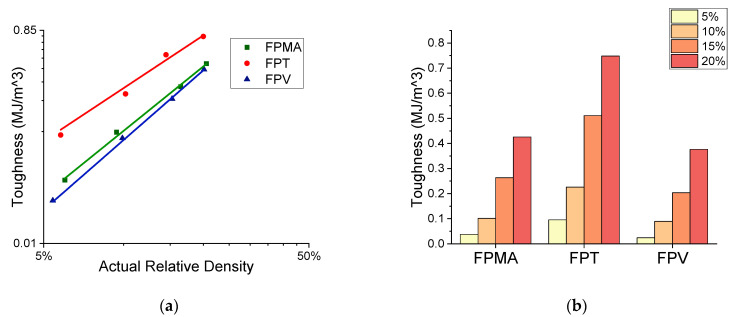
Deduced mechanical properties, (**a**) toughness vs. relative density, (**b**) toughness histogram.

Figure 9a,b shows the toughness at different relative densities. Toughness (EA) was calculated as the total area under the stress–strain curve up to 60% strain or the integral of the stress–strain curve shown in Equation (1) below, where ε is the strain and σ is the stress [58].

(3) $$EA\left(\varepsilon \right)=\overset{\varepsilon }{\underset{0}{\int }}\sigma \left(\varepsilon \right)d\varepsilon$$

The results of the power-law fitting are presented in Table 3.

The exponent (n) of the power-law fit provides useful insight into the lattices’ mechanical properties and behaviors. The FPT has an n of 1.16, indicating a stretching-dominated behavior that is efficient at load bearing, making its stiffness least affected by a change in relative density between all the three novel lattices. The FPV, on the other hand, has an n of 3.59, indicating a bending-dominated behavior, and with the highest power-law exponent, sees the greatest change in stiffness for a change in relative density. The FPMA shows a near stretching dominated behavior with an n of 1.30. As for the peak stresses, the FPV also has the highest n exponent, giving rise to a large change in specific energy absorption with a change in volume fraction. The FPMA and FPT scale relatively similarly with changes in the relative density. The FPMA and FPT showed similar scaling with the yield strength, with the FPV having again the largest n. With regard to the toughness, the FPT exhibits a superior performance, surpassing both the FPV and the FPMA at all four tested relative densities, with only a minor sacrifice in peak stress and modulus.

**Figure 10:**
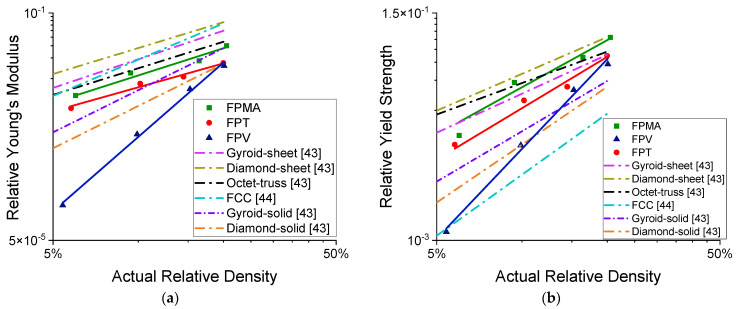
(**a**) Relative modulus vs. relative density, (**b**) relative yield strength vs. relative density.

Figure 10 presents the relative modulus and relative yield strength of the novel lattices alongside the lattices found in literature. The results obtained have been fitted to a power-law relation. For the modulus, the power law is of the form $\frac{E}{E_{s}}=C_{1}\left(\rho_{}^{n}\right)$, where *E* is the lattice’s modulus, and *E*_s_ is the parent materials modulus. While for the yield strength, the power law is of the form $\frac{\sigma_{yl}}{\sigma_{ys}}=C_{2}\left(\rho_{}^{m}\right)$, where $\sigma_{yl}$ is the yield strength of the lattice and $\sigma_{ys}$ is the yield strength of the parent material. The powers *n* and *m* are the same as given in Table 3. However, the coefficient $C_{1}=0.25,\;0.12,\;6.11$ and $C_{2}=0.92,\;0.79,5.34$ are given for FPMA, FPT, and FPV, respectively. Although the FPV is on the lower side of Figure 10a, the FPT and FPMA have moduli comparable to TPMS-based lattices and are close to truss lattices. As for the relative yield strength, the novel lattices surpass most of the presented lattices from the literature, except for the sheet TPMS-based lattices.

**Figure 11:**
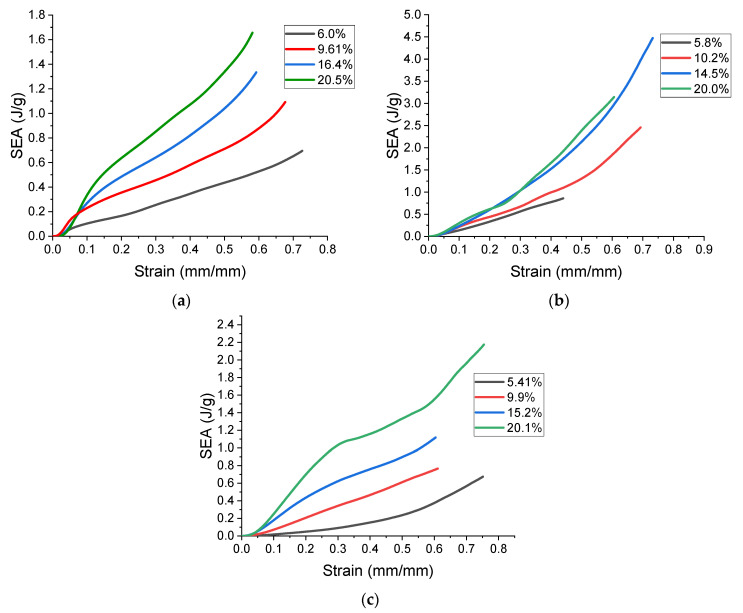
Specific energy absorption vs. strain, (**a**) flat-plate modified auxetic, (**b**) flat-plate tesseract, (**c**) flat plate vintile.

The specific energy absorption (SEA) vs. strain is plotted in Figure 11, and it was found by dividing the area under the stress–strain curve by the lattice’s density (ρ^*^), as shown in the equation below, where ($\varepsilon_{d}$) is the densification strain [58].

(4) $$SEA=\frac{\int_{0}^{\varepsilon_{d}}\sigma \left(\varepsilon \right)d\varepsilon }{\rho^{*}}$$

The FPT can reach a remarkable SEA of 4.50 J/g at a strain of 0.7, the FPV reaches a SEA of 2.20 J/g at a strain of 0.75, and the MA reaches an SEA of 1.70 J/g at a strain of 0.58. However, it is worth noting that the FPT at 20% relative density sees a decrease in its SEA due to the early onset of densification. It is interesting to note that the effects of cell architecture become less pronounced with an increase in relative density, as evident by Figure 8, where the fits tend to converge to a single point. However, that does not seem to be the case for toughness, bolstering that the FPT has the best energy absorption regardless of the relative density. This remarkable energy absorption ability makes the FPT a suitable candidate for lightweight energy absorption applications, such as those commonly required in aerospace load-bearing structures.

**Figure 12:**
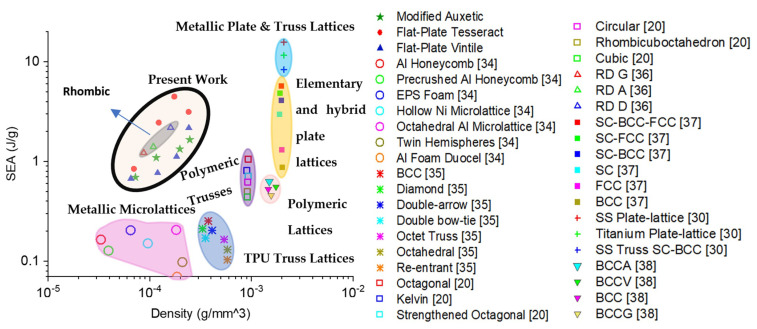
Ashby plot of specific energy absorption vs. density.

Figure 12 presents the specific energy absorption of the three novel lattices designed in this work with some of the lattices found in the literature. It can be clearly seen that even with such a low density, the lattices presented exhibit specific energy absorption values that surpass most of the shown lattices and are closest in effectiveness to metallic plate lattices (≈3.8 J/g difference), with a density almost 20 times less. This further illustrates the efficacy of the proposed lattice designs for energy absorption applications, particularly the flat-plate tesseract.

## 5. Conclusions

In conclusion, three lattices with novel architectures were designed, fabricated, and tested under quasi-static compression. The Formlabs Durable Resin was used to fabricate the novel lattices, and the mechanical properties of the resin were experimentally obtained. Using the obtained data, a finite element-based homogenization was performed to determine the lattices’ anisotropy. The anisotropic analysis showed that the anisotropy index of FPV is highly dependent on relative density. After quasi-static compression testing, the behavior of the different lattices was analyzed and discussed, and their mechanical properties were deduced. The flat-plate modified auxetic lattice had the highest stiffness and showed mixed-bending and stretching behavior, with failure being initiated by buckling of the unit cell members. The flat-plate vintile showed a ductile failure mode at lower volume fractions, with a transition to a brittle failure mode at higher volume fractions. Lastly, the flat-plate tesseract showed remarkable stiffness and had the highest energy absorption of all the three novel lattices, and whose specific energy absorption surpassed many lattices found in literature.

## Figures and Tables

**Table 1:** Characteristics of architected unit cell and lattice structure.

Type	Unit Cell Design	Unit Cell Parameters		2 × 2 × 2 Lattice Structure	Printed Specimen
**Flat-Plate Modified Auxetic**	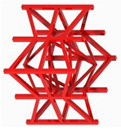	ρ=0.049 ρ=0.099 ρ=0.151 ρ=0.201	$t_{min}=0.90\;\mathrm{mm}$ $t_{min}=1.25\;\mathrm{mm}$ $t_{min}=1.75\;\mathrm{mm}$ $t_{min}=2.00\;\mathrm{mm}$	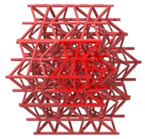	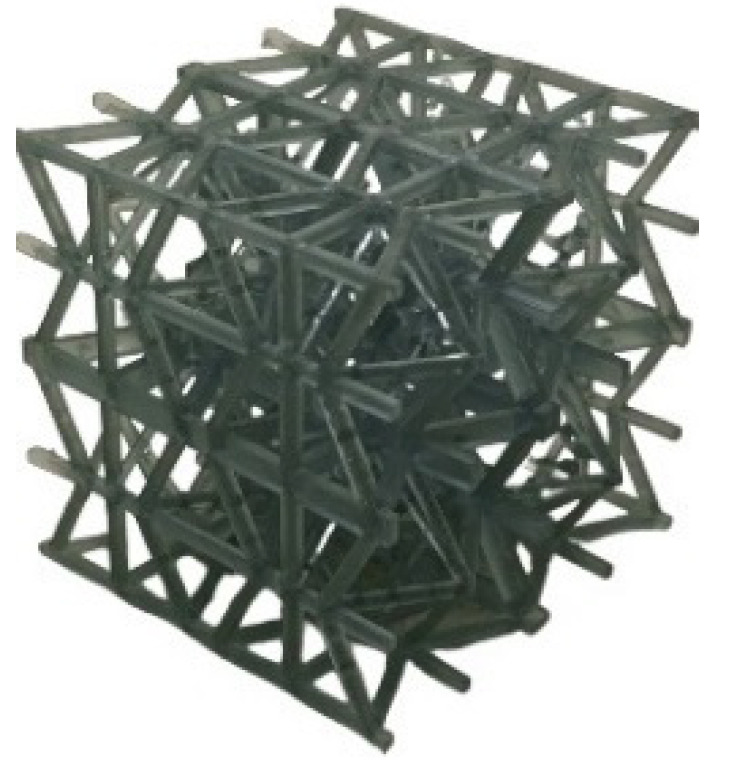
**Flat-Plate Tesseract**	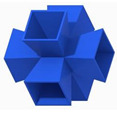	ρ=0.052 ρ=0.099 ρ=0.148 ρ=0.203	$t_{min}=0.40\;\mathrm{mm}$ $t_{min}=0.65\;\mathrm{mm}$ $t_{min}=1.00\;\mathrm{mm}$ $t_{min}=1.90\;\mathrm{mm}$	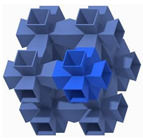	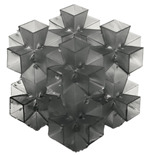
**Flat-Plate Vintile**	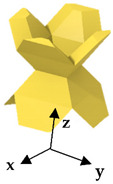	ρ=0.052 ρ=0.098 ρ=0.152 ρ=0.199	$t_{min}=0.60\;\mathrm{mm}$ $t_{min}=1.15\;\mathrm{mm}$ $t_{min}=1.75\;\mathrm{mm}$ $t_{min}=2.50\;\mathrm{mm}$	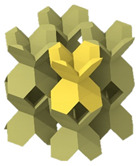	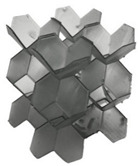

**Table 2:** Lattices at different strains.

Architecture/Strain	0.1	0.2	0.3	0.4	0.5
(a) FPMA—15%	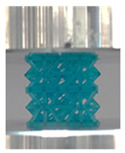	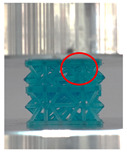	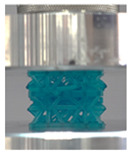	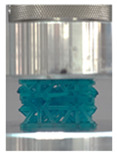	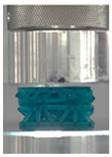
(b) FPV—5%	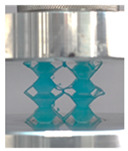	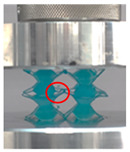	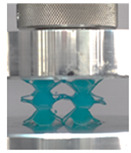	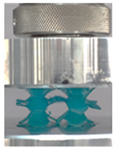	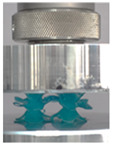
(c) FPV—20%	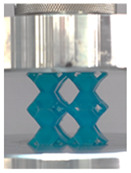	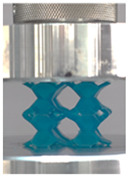	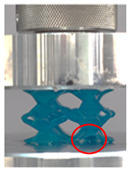	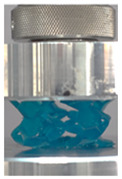	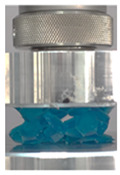
(d) FPT—5%	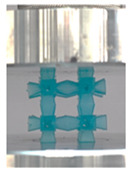	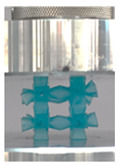	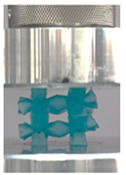	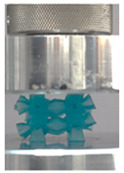	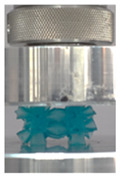
(e) FPT—10%	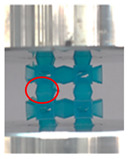	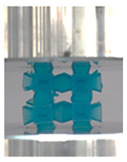	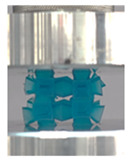	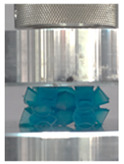	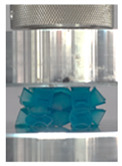
(f) FPT—20%	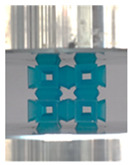	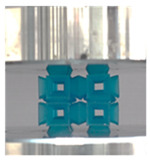	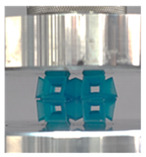	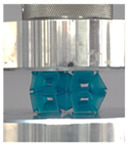	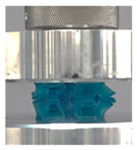

**Table 3:** Power-law fitting constants.

	Compressive Modulus (MPa)		Peak Stress (MPa)		Toughness (MJ/m^3^)		Yield Strength (KPa)	
	C	n	C	n	C	n	C	n
FPMA	275.40	1.30	18.74	1.65	9.11	1.94	12,196.07	1.50
FPT	131.17	1.16	11.38	1.51	9.87	1.59	10,477.49	1.63
FPV	6716.66	3.59	92.13	2.75	10.85	2.10	70,898.04	2.85

## Data Availability

All data and models used during the study appear in the submitted article.
